# Use of a genetically engineered mouse model as a preclinical tool for HER2 breast cancer

**DOI:** 10.1242/dmm.023143

**Published:** 2016-02-01

**Authors:** Helen Creedon, Lucy A. Balderstone, Morwenna Muir, Jozef Balla, Laura Gomez-Cuadrado, Natasha Tracey, Joseph Loane, Teresa Klinowska, William J. Muller, Valerie G. Brunton

**Affiliations:** 1 Edinburgh Cancer Research UK Centre, University of Edinburgh, Crewe Road South, Edinburgh EH4 2XR, UK; 2 Pathology Department, Western General Hospital, Edinburgh EH4 2ZD, UK; 3 AstraZeneca Oncology iMed, Alderley Park, Macclesfield SK10 4TG, UK; 4 Goodman Cancer Research Center, McGill University, Montreal, CanadaH3A 1A3

**Keywords:** HER2, Breast cancer, Resistance, Epithelial-to-mesenchymal transition

## Abstract

Resistance to human epidermal growth factor receptor 2 (HER2)-targeted therapies presents a major clinical problem. Although preclinical studies have identified a number of possible mechanisms, clinical validation has been difficult. This is most likely to reflect the reliance on cell-line models that do not recapitulate the complexity and heterogeneity seen in human tumours. Here, we show the utility of a genetically engineered mouse model of HER2-driven breast cancer (MMTV-NIC) to define mechanisms of resistance to the pan-HER family inhibitor AZD8931. Genetic manipulation of MMTV-NIC mice demonstrated that loss of phosphatase and tensin homologue (PTEN) conferred *de novo* resistance to AZD8931, and a tumour fragment transplantation model was established to assess mechanisms of acquired resistance. Using this approach, 50% of tumours developed resistance to AZD8931. Analysis of the resistant tumours showed two distinct patterns of resistance: tumours in which reduced membranous HER2 expression was associated with an epithelial-to-mesenchymal transition (EMT) and resistant tumours that retained HER2 expression and an epithelial morphology. The plasticity of the EMT phenotype was demonstrated upon re-implantation of resistant tumours that then showed a mixed epithelial and mesenchymal phenotype. Further AZD8931 treatment resulted in the generation of secondary resistant tumours that again had either undergone EMT or retained their original epithelial morphology. The data provide a strong rationale for basing therapeutic decisions on the biology of the individual resistant tumour, which can be very different from that of the primary tumour and will be specific to individual patients.

## Abstract (precis)

**Editors' choice:** This study demonstrates the utility of a transgenic mouse model of breast cancer to define mechanisms of resistance to a pan-HER family inhibitor and identifies a link with EMT.

## INTRODUCTION

Human epidermal growth factor receptor 2 (HER2) gene amplification and/or protein overexpression occurs in around 20% of breast cancers and is associated with poor prognosis. Several drugs capable of specifically targeting the HER2 pathway have been developed for use in both early and late HER2-positive disease and have had a significant impact on the treatment of HER2-positive breast cancer (Arteaga et al., 2012); these include antibodies directed against HER2, such as trastuzumab and pertuzumab, and small molecule tyrosine kinase inhibitors that target the kinase activity of HER2 and HER1, such as lapatinib. Although initial response rates to the current HER2-targeted therapies are good, resistance is inevitable. Further tyrosine kinase inhibitors, including AZD8931 (sapatinib) and neratinib, have been developed in an attempt to improve efficacy rates and the duration of response. Preclinical studies have identified numerous mechanisms of both *de novo* and acquired resistance (Creedon et al., 2014; Rexer and Arteaga, 2012), although their clinical validation has been more difficult, which reflects the inability of the conventional cell-based approaches to model the complexity of the human disease adequately.

The limitation of conventional cell culture and mouse xenograft studies is well recognized as an obstacle to the effective translation of preclinical findings into clinical benefit (Sharpless and Depinho, 2006). Use of genetically engineered models in which tumours develop *in situ* in the context of an intact microenvironment is a viable alternative for preclinical assessment of both drug response and mechanisms of resistance (van Miltenburg and Jonkers, 2012). Generation of autochthonous tumours driven by cell-specific expression of oncogenic drivers or loss of tumour suppressors relevant to human tumours gives rise to tumours in which the histopathology and disease progression also recapitulate many aspects of the human disease, providing more relevant models with which to study drug response.

Here, we describe the use of a HER2-driven model of mammary tumorigenesis as a preclinical tool to study response and resistance mechanisms in HER2-positive breast cancer. We have used the MMTV-NIC (Neu-IRES-Cre) model (Ursini-Siegel et al., 2008), which employs a bicistronic transcript to co-express activated ErbB2/Neu (HER2) with MMTV-Cre recombinase, resulting in the formation of activated ErbB2/Neu-driven mammary tumours. The advantage of this model is that the coupling of activated ErbB2/Neu with Cre recombinase in the same cell means that Cre-negative tumour cells are not generated, allowing the efficient Cre-mediated deletion of additional conditional alleles (Schade et al., 2009). This allows validation of potential mechanisms of *de novo* resistance, such as loss of phosphatase and tensin homologue (PTEN). Loss of PTEN and subsequent activation of the phosphoinositide 3-kinase (PI3K) pathway has been identified as a key determinant of trastuzumab sensitivity and has been associated with poorer overall survival in trastuzumab-treated patients (Berns et al., 2007; Esteva et al., 2010; Nagata et al., 2004), although the impact on lapatinib resistance remains unclear (Xia et al., 2007). Here, we have coupled loss of PTEN with HER2 activation in the MMTV-NIC model and demonstrate that loss of PTEN is associated with *de novo* resistance to the small molecule tyrosine kinase inhibitor sapatinib (AZD8931). We also show the utility of the model for identifying mechanisms of acquired resistance to HER2-targeted therapy and identify the induction of an epithelial-to-mesenchymal transition (EMT) in a subpopulation of AZD8931-resistant tumours.

## RESULTS

### Loss of PTEN confers resistance to AZD8931

Initial experiments were carried out to determine whether the MMTV-NIC model was sensitive to the HER family tyrosine kinase inhibitor AZD8931. As loss of PTEN and activation of the PI3K signalling pathway have previously been reported to confer resistance to trastuzumab, cohorts of both MMTV-NIC PTEN^+/+^ and MMTV-NIC PTEN^+/−^ mice were used. Both MMTV-NIC PTEN^+/+^ and MMTV-NIC PTEN^+/−^ mice developed on average four tumours per mouse with 100% penetrance. As described previously, loss of PTEN accelerated tumour onset in the MMTV-NIC mice (Schade et al., 2009). The median age of tumour onset was 102 days in the MMTV-NIC PTEN^+/−^ cohort compared with 150 days in the MMTV-NIC PTEN^+/+^ cohort (*P*=0.0001, Gehan–Breslow–Wilcoxon test; Fig. S1A). Western blot analysis of tumours showed reduced expression of PTEN and increased phosphorylation of Akt in tumours taken from the MMTV-NIC PTEN^+/−^ mice, consistent with increased signalling through the PI3K pathway (Fig. S1B).

**Fig. 1.**
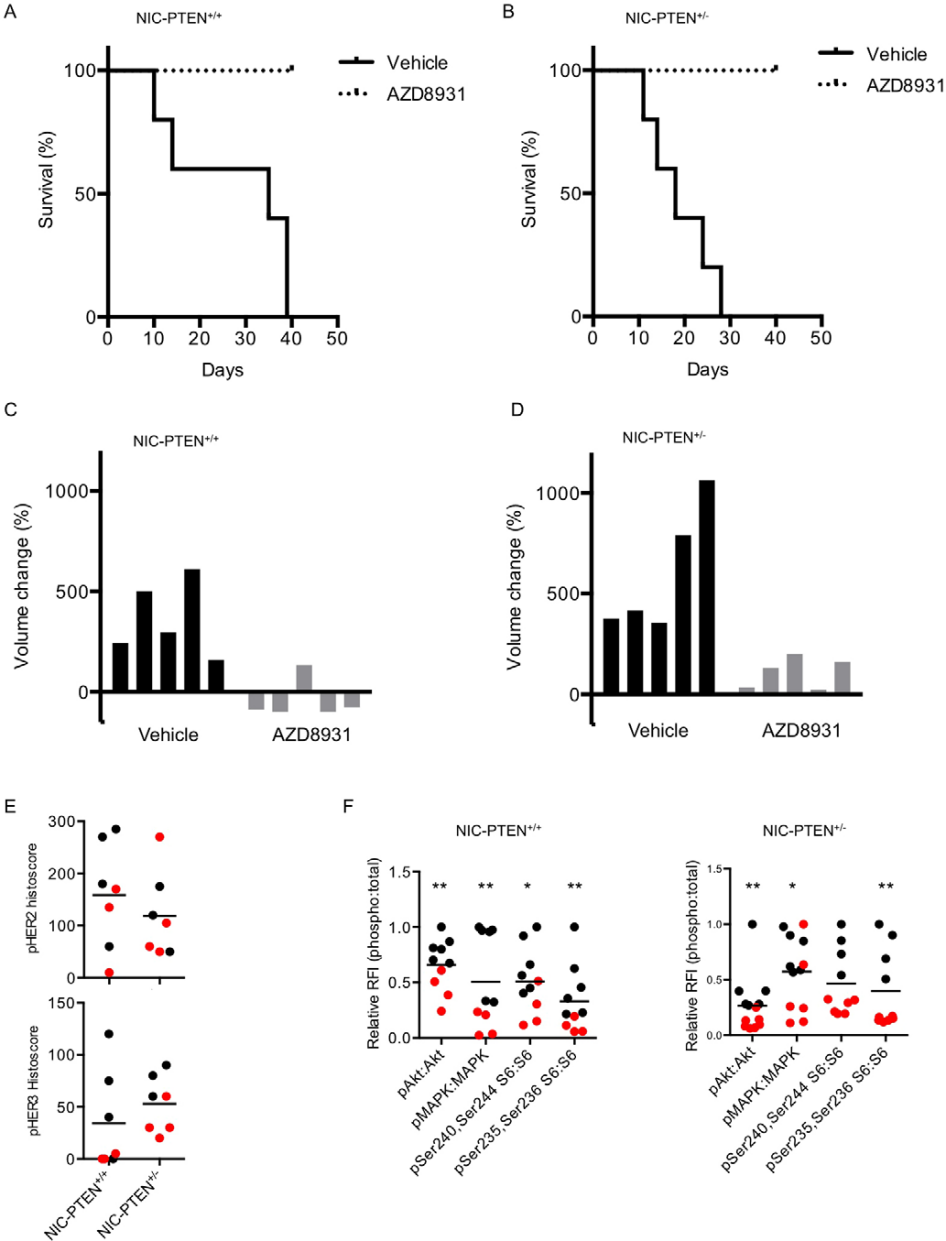
**PTEN status determines the sensitivity to AZD8931.** Cohorts of MMTV-NIC-PTEN^+/+^ and NIC-PTEN^+/−^ mice were randomized to treatment with daily AZD8931 or vehicle, and the tumour response was monitored. (A) Overall survival in vehicle- (*n*=5) and AZD8931-treated (*n*=5) NIC-PTEN^+/+^ mice (*P*=0.0043, Gehan–Breslow–Wilcoxon test). (B) Overall survival in vehicle- (*n*=5) and AZD8931-treated (*n*=5) NIC-PTEN^+/−^ mice (*P*=0.0039, Gehan–Breslow–Wilcoxon test). (C) Waterfall plot of percentage tumour volume change over duration of experiment in NIC-PTEN^+/+^ vehicle- and AZD8931-treated animals (*P*=0.0079, Mann–Whitney *U*-test). (D) Waterfall plot of percentage tumour volume change over duration of experiment in NIC-PTEN^+/−^ vehicle- and AZD8931-treated animals (*P*=0.0079, Mann–Whitney *U*-test). Vehicle-treated mice were sacrificed when the tumour burden reached the maximal permitted size, at 10-39 days for MMTV-NIC PTEN^+/+^ mice and 11-24 days for MMTV-NIC PTEN^+/−^ mice. AZD8931 treatment was stopped at 40 days, when all vehicle-treated animals were sacrificed because of tumour burden. (E) Immunohistochemical analysis was performed on paraffin-embedded sections of AZD8931- and vehicle-treated tumours with pTyr 1221/1222 HER2 and pTyr 1289 HER3 antibodies. Membranous histoscore calculated as the sum of the product of the percentage of cells stained by the intensity graded from 0 to 3, where 1=weak, 2=moderate and 3=strong staining [histoscore=(% *1)+(% *2)+(% *3)]. pTyr 1221/1222 HER2 staining: NIC-PTEN^+/+^, *P*=0.48; NIC-PTEN^+/−^, *P*=1.0; and pTyr1289 HER3 staining: NIC-PTEN^+/+^, *P*=0.20; NIC-PTEN^+/−^, *P*=0.07; Mann–Whitney *U*-test comparing vehicle- and AZD8931-treated tumours. Black data points represent vehicle-treated tumours. Red data points represent AZD8931-treated tumours. Bars represent mean value for each genotype. (F) Reverse-phase protein array analysis was performed on lysate from AZD8931- (red data points) and vehicle-treated (black data points) NIC-PTEN^+/+^ and NIC-PTEN^+/−^ tumours. The ratio of phospho:total protein relative fluorescence intensity (RFI) value is presented and normalized to the maximal value in each data set. *P≤0.05 and **P≤0.01 comparing vehicle and AZD8931 for each antibody, Mann–Whitney *U*-test.

To assess the response to AZD8931, we randomized cohorts of MMTV-NIC PTEN^+/+^ and MMTV-NIC PTEN^+/−^ mice to treatment with either AZD8931 or vehicle. Median survival in the vehicle arm of the MMTV-NIC PTEN^+/+^ cohort after the start of treatment was 35 days (range 10-39 days), compared with 18 days (range 11-24 days) in vehicle-treated MMTV-NIC PTEN^+/−^ mice. Drug treatment was stopped at 40 days, when all vehicle-treated animals were sacrificed because of tumour burden. At this time, none of the drug-treated animals had to be sacrificed because of tumour burden (Fig. 1A,B). When we looked at the growth of the individual index tumours (defined as the largest tumour at the time of sacrifice) in the different cohorts after 40 days, we saw that all AZD8931-treated MMTV-NIC PTEN^+/+^ tumours initially responded to treatment and two out of five tumours fully resolved. The growth of a further two tumours was inhibited, whereas the final tumour initially responded but after 17 days of drug treatment became insensitive, and after 40 days of treatment its volume had increased by 134.2%. By comparison, the tumours in the vehicle-treated arm continued to grow throughout the experiment, and the median percentage change in tumour volume was an increase of 294.6% (Fig. 1C). By contrast, all but one of five AZD8931-treated MMTV-NIC PTEN^+/−^ tumours became rapidly insensitive to AZD8931, and by day 40 all AZD8931-treated tumours had grown beyond their initial starting volume, with a median percentage change in tumour volume of 131.1%. As expected, all vehicle-treated tumours continued to grow steadily throughout the experiment, with a median percentage change in tumour volume of 415.1% (Fig. 1D). In summary, AZD8931 resulted in tumour shrinkage in the majority of MMTV-NIC PTEN^+/+^ animals, but although it slowed tumour growth in MMTV-NIC PTEN^+/−^ animals it did not cause tumour resolution. We also noted that by day 40 there were fewer additional tumours in both the MMTV-NIC PTEN^+/+^ and MMTV-NIC PTEN^+/−^ animals treated with AZD8931, and although this did not reach statistical significance the reduction in tumour burden was greater in the MMTV-NIC PTEN^+/+^ mice, consistent with the increased sensitivity of the MMTV-NIC PTEN^+/+^ tumours to AZD8931 (median values for tumours per mouse: MMTV-NIC PTEN^+/+^, vehicle=5; MMTV-NIC PTEN^+/+^, AZD8931=1; MMTV-NIC PTEN^+/−^, vehicle=6; MMTV-NIC PTEN^+/−^, AZD8931=3; *P*=0.1025, Kruskal–Wallis). This illustrates the significant heterogeneity in response to AZD8931 between the two different cohorts and demonstrates that loss of PTEN, leading to activation of the PI3K pathway, confers *de novo* resistance to AZD8931.

As there was little residual tissue from the AZD8931-treated MMTV-NIC PTEN^+/+^ animals at the completion of the experiment, additional cohorts of both MMTV-NIC PTEN^+/+^ and MMTV-NIC PTEN^+/−^ mice were treated with vehicle or AZD8931 for 3 days and effects on HER family signalling pathways assessed. There was a reduction in phosphorylation of HER2 and HER3 in MMTV-NIC PTEN^+/+^ and MMTV-NIC PTEN^+/−^ tumours following treatment with AZD8931 compared with vehicle-treated animals, although this did not reach statistical significance after histoscoring (Fig. 1E). This reflects the heterogeneous expression and activation of both HER2 and HER3 in the tumours (Fig. S1C). No significant epidermal growth factor receptor (EGFR/HER1) expression was detected in the MMTV-NIC tumours, so it was not possible to assess effects on EGFR activation (results not shown). We used reverse-phase protein arrays to look at downstream signalling to Akt, mitogen-activated protein kinase (MAPK) and S6 in the AZD8931-treated tumours and found that their activation was significantly inhibited in MMTV-NIC PTEN^+/+^ and MMTV-NIC PTEN^+/−^ tumours (Fig. 1F). Immunohistochemical analysis showed that pAkt was confined to the tumour cells and not expressed in the surrounding stroma, whereas pMAPK was also expressed in the stroma, and the reduced expression of pMAPK might therefore also reflect reduced activation of MAPK in the surrounding stroma (Fig. S1D). Thus short-term treatment with AZD8931 inhibits HER family signalling in both tumour types, and therefore, the differential response of the MMTV-NIC PTEN^+/+^ and MMTV-NIC PTEN^+/−^ tumours does not reflect an inability of the drug to inhibit the target in the different tumours.

### Establishment of orthotopic transplanted tumours

**Fig. 2.**
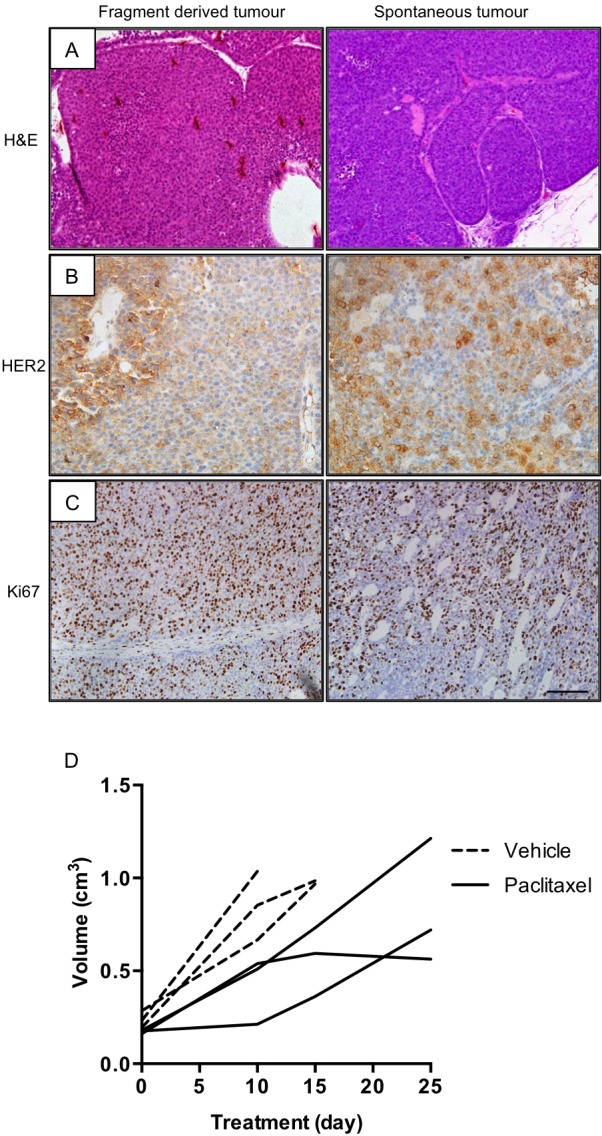
**Transplantation of NIC-PTEN^+/−^ fragments generated tumours that were indistinguishable from the parental tumours.** (A) Representative H&E images of fragment-derived and spontaneous tumours from NIC-PTEN^+/−^ mice. (B,C) Representative images of HER2 (B) and Ki67 (C) expression in fragment-derived and spontaneous tumours. Scale bar: 100 µm. (D) Growth rate of vehicle- (*n*=3) and paclitaxel-treated (*n*=3) fragment-derived tumours.

The multifocal nature of the MMTV-NIC model means that it is not possible to study mechanisms of acquired drug resistance. We therefore established whether the MMTV-NIC PTEN tumours could be orthotopically transplanted into syngeneic wild-type FVB/N mice to provide a more tractable model for drug-resistance studies. We were able to establish tumours after transplantation of tumour fragments from MMTV-NIC PTEN^+/−^ tumours but not from MMTV-NIC PTEN^+/+^ tumour fragments. Examination of sections stained with haematoxylin and eosin (H&E) from the fragment-derived MMTV-NIC PTEN^+/−^ tumours confirmed the presence of highly mitotic, grade 3 carcinomas, which were indistinguishable from tumours that developed in the parental MMTV-NIC PTEN^+/−^ model (Fig. 2A). Both parental and fragment-derived tumours demonstrated inter-and intratumoral heterogeneity in HER2 expression (Fig. 2B) and, consistent with the frequent observation of mitotic figures on H&E sections, a high percentage of nuclei stained positively for Ki67 in both parental and fragment-derived tumours (Fig. 2C). The orthotopic transplantation model therefore provides a useful tool by circumventing problems associated with the multifocal nature of the genetically engineered model.

Initial studies to determine the suitability of the MMTV-NIC transplantation model for drug-efficacy studies were carried out using the taxane paclitaxel, whose role in the management of both early and metastatic breast cancer is well established (Gajria et al., 2010; Ghersi et al., 2005). Paclitaxel treatment resulted in a statistically significant increase in overall survival of mice bearing fragment-derived tumours. Median overall survival was increased from 14 days in vehicle-treated animals (range 7-14 days) to 24 days (range 24-24 days) in drug-treated animals (*P*=0.03, Gehan–Breslow–Wilcoxon test). Looking at the response of individual tumours to treatment, we found that although paclitaxel slowed tumour growth, it did not result in tumour shrinkage (Fig. 2D).

### Development of resistance to AZD8931

**Fig. 3.**
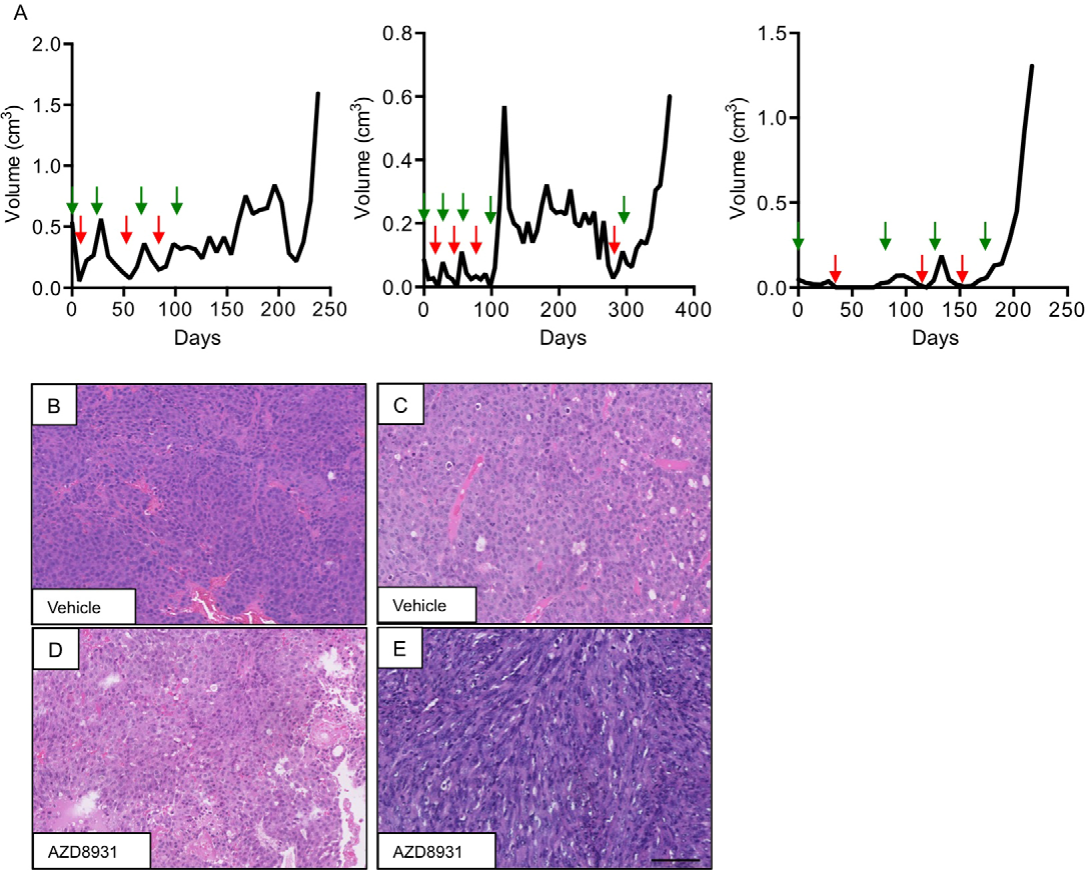
**Generation of fragment-derived tumours with acquired resistance to AZD8931.** (A) Representative growth curves of three independent NIC-PTEN^+/**−**^ tumour fragments treated with AZD8931. Repeated cycles of AZD8931 were administered to facilitate the selection of tumours with acquired resistance to AZD8931. Green arrows indicate the start of treatment and red arrows indicate when treatment was stopped. (B,C) Representative H&E images of AZD8931-naïve (vehicle) and AZD8931-resistant tumours. (D) AZD8931-resistant tumour phenotypically indistinguishable from AZD8931-naïve tumour. (E) AZD8931-resistant tumour consisting of spindle-shaped cells. Scale bar: 100 µm.

We next determined whether the MMTV-NIC tumours could be used to model acquired resistance to AZD8931. We generated tumour fragments from three MMTV-NIC PTEN^+/−^ donor mice. From each mouse, donor tumour fragments were then transplanted into cohorts of six wild-type FVB/N mice. After the development of established tumours, mice were randomized to treatment with either vehicle (*n*=3) or AZD8931 (*n*=3). AZD8931 treatment resulted in an initial inhibition of tumour growth in all mice. When tumours had regressed to ≤0.1 cm^3^, AZD8931 treatment was stopped. After the subsequent regrowth of the tumour, treatment was then resumed when tumours reached ≥0.3 cm^3^. This cycle was repeated until tumours either resolved or became resistant and were able to continue growing in the presence of ongoing treatment. The resistant tumours originated from different donors, and the duration of treatment required for the individual tumours to become resistant varied between individual tumours (Fig. 3A).

Histopathological examination of the matched AZD8931-naïve and -resistant tumours revealed that although all the AZD8931-naïve tumours were histologically indistinguishable from each other (Fig. 3B,C) there were significant differences in the AZD8931-resistant tumours (Fig. 3D,E). Although some of the resistant tumours had the same histopathological phenotype as their matched naïve tumour (Fig. 3D), a subset of resistant tumours had a more pleomorphic appearance and were composed of spindle cells, suggestive of tumours undergoing EMT (Fig. 3E).

**Fig. 4.**
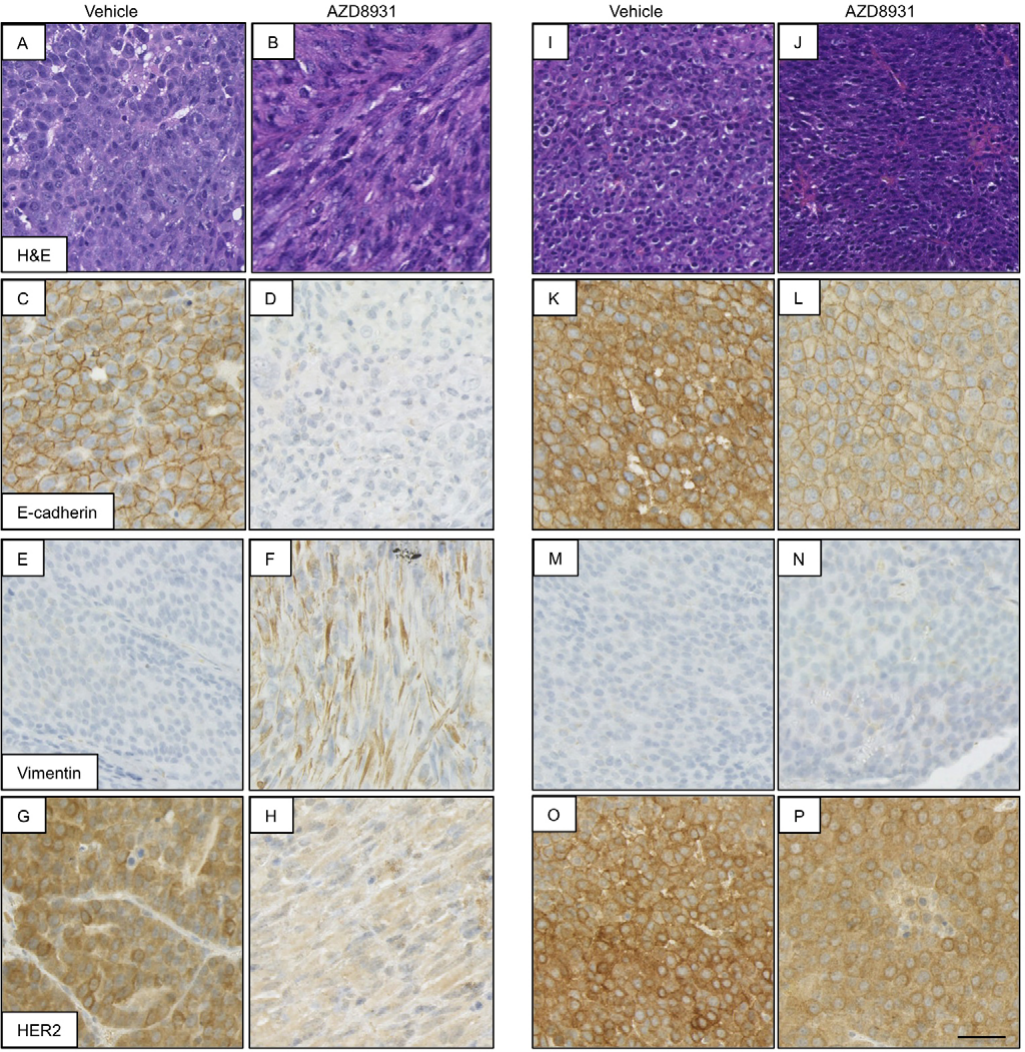
**AZD8931 resistance is associated with EMT in a subpopulation of tumours.** Analysis of AZD8931-naïve (vehicle) and AZD8931-resistant tumours showing representative images of H&E staining and immunohistochemical analysis of E-cadherin, vimentin and HER2. Scale bar: 50 µm. (A-H) AZD8931-resistant spindle cell tumour and corresponding vehicle-treated tumour. (I-P) AZD8931-resistant tumour phenotypically indistinguishable from AZD8931-naïve (vehicle) tumour and corresponding vehicle-treated tumour.

Loss of cell surface E-cadherin is an established marker of EMT, and although strong membranous expression of E-cadherin was seen in all drug-naïve tumours, loss of E-cadherin in the resistant tumours was associated with the conversion to a spindle cell morphology (Fig. 4A-D), with the resistant tumours that had not undergone the morphological change retaining expression of E-cadherin (Fig. 4I-L). This loss of E-cadherin was accompanied by expression of the mesenchymal marker vimentin in the resistant spindle cell tumours (Fig. 4E,F), indicating that these resistant tumours had undergone EMT. HER2 expression was preserved in the resistant tumours that had retained the histopathological features of the drug-naïve tumours (Fig. 4O,P), but total loss of membranous HER2 expression was seen in the resistant spindle cell tumours (Fig. 4G,H).

**Fig. 5.**
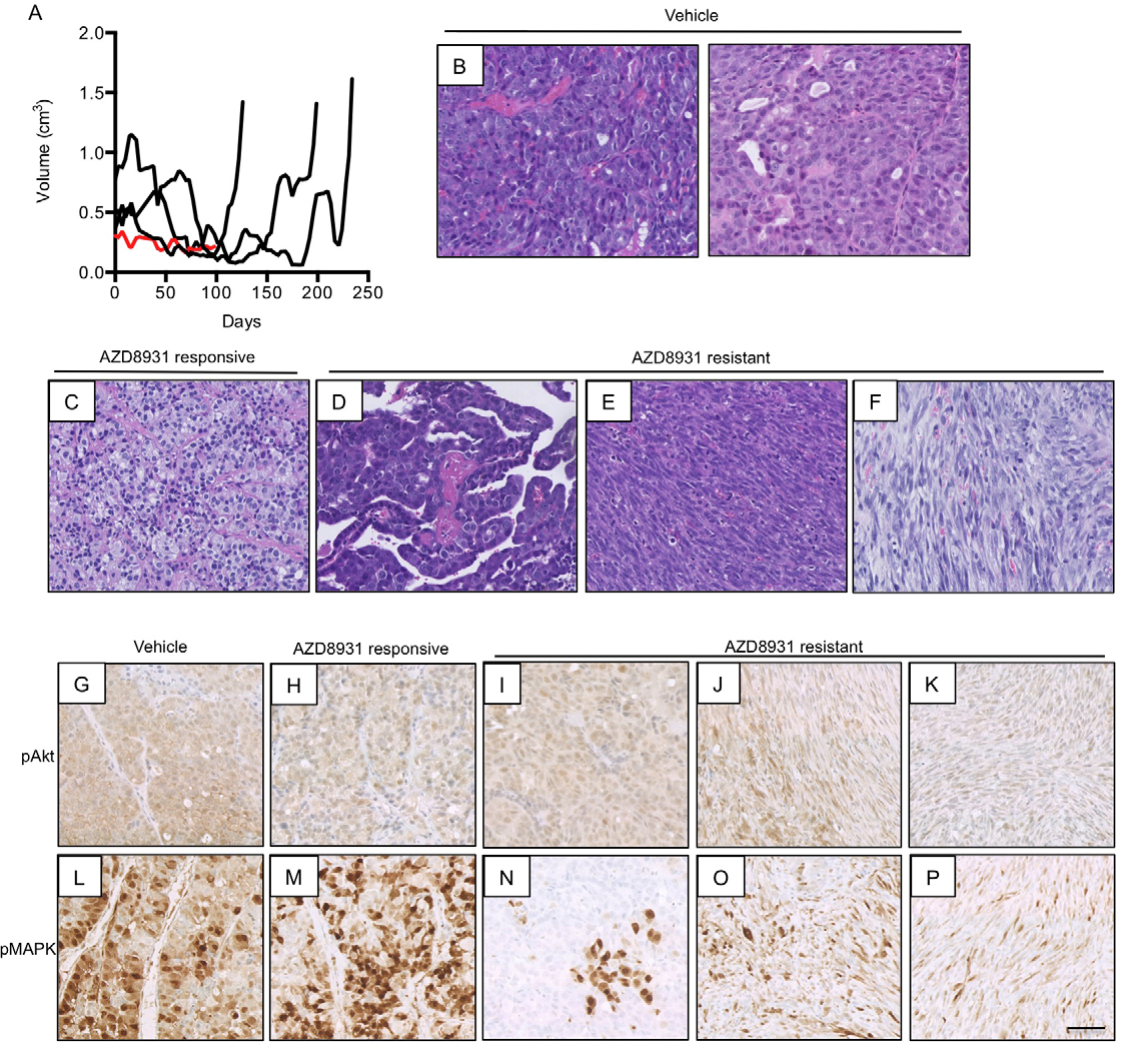
**Generation of secondary resistance in NIC-PTEN^+/−^ tumour fragments.** (A) Growth curves of NIC-PTEN^+/−^ AZD8931-resistant tumour fragments following treatment with repeated cycles of AZD8931. Black lines represent tumours that developed resistance to AZD8931. Red line represents tumour with growth inhibited by AZD8931. Treatment was stopped after 90 days because of lack of tumour growth. (B) Representative H&E staining of vehicle-treated tumours. (C-F) H&E staining of AZD8931-treated tumours. (C) AZD8931-responsive tumour (red line in A) with growth inhibited by AZD893. (D) AZD8931-resistant tumour that has retained the morphology of the AZD8931-naïve (vehicle) tumours. (E,F) AZD8931-resistant tumours with a spindle cell morphology. (G-P) Immunohistochemical analysis of pAkt (G-K) and pMAPK (L-P) in vehicle- and AZD8931-treated tumours. Scale bar: 50 µm.

To establish whether the resistant phenotype was stable, fragments from one individual resistant tumour were re-implanted into cohorts of wild-type FVB/N mice. After the development of established tumours, mice were randomized to treatment with either vehicle or AZD8931. AZD8931 treatment prevented the growth of one tumour, although tumour regression did not occur (Fig. 5A, red growth curve, ‘AZD8931 responsive’). The remaining three tumours responded initially to AZD8931, although resistance did develop over time (Fig. 5A, black growth curves, ‘AZD8931 resistant’). The onset of secondary resistance was faster than in the original drug-naïve tumour fragments (compare Figs 3A and 5A).

The vehicle-treated tumours were all highly mitotic, containing rounded cells with pleomorphic nuclei (Fig. 5B). The tumour whose growth was inhibited by AZD8931 had mild to moderately pleomorphic nuclei and no mitoses (Fig. 5C). The resistant tumours had distinct morphologies; two of the tumours were composed of highly mitotic spindle cells with similar histology to those seen in the primary resistant tumours that had undergone EMT (compare Fig. 5E,F with Fig. 3C), whereas the remaining resistant tumour had a papillary architecture with moderate/marked nuclear pleomorphism (Fig. 5D). As we had previously demonstrated a reduction in pAkt and pMAPK after acute treatment with AZD8931 (Fig. 1F), we looked at activation of Akt and MAPK following development of resistance to AZD8931 (Fig. 5G-P). Chronic exposure to AZD8931 did not reduce pAkt and pMAPK levels, although one AZD89231 resistant tumour showed an overall reduction in pMAPK staining, with only small pockets of pMAPK-positive cells scattered throughout the tumour (Fig. 5N).

**Fig. 6.**
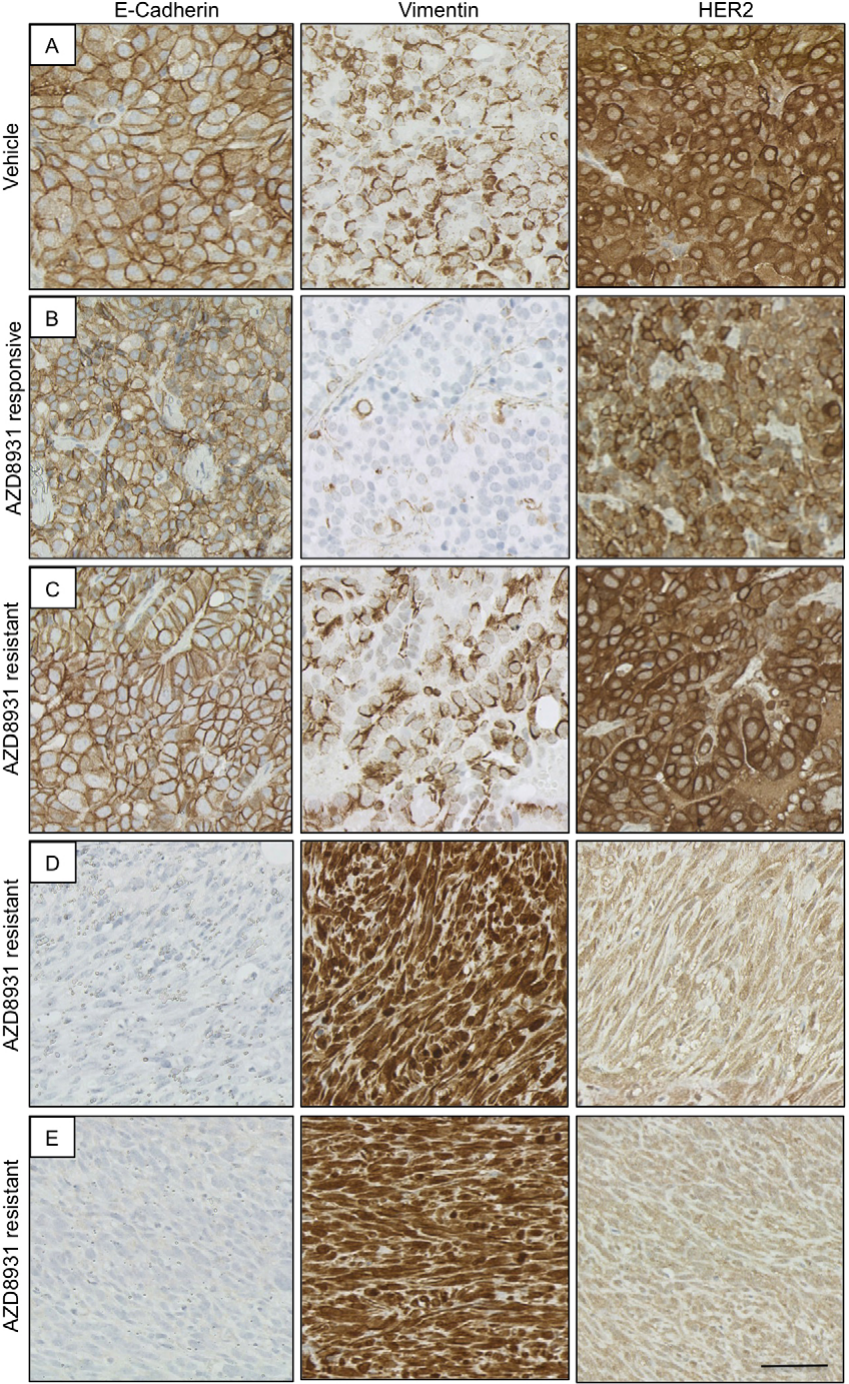
**Development of EMT in AZD8931-resistant tumours.** Immunohistochemical analysis of E-cadherin, vimentin and HER2 in (A) vehicle- and (B-E) AZD8931-treated tumours. (B) AZD8931-responsive tumour with growth inhibited by AZD8931. (C) AZD8931-resistant tumour that has retained the morphology of the AZD8931-naïve (vehicle) tumours. (D,E) AZD8931-resistant tumours with a spindle cell morphology. Scale bar: 50 µm.

Further analysis of the tumours revealed that all vehicle-treated tumours expressed both E-cadherin and vimentin (Fig. 6A). The AZD8931-responsive tumour was also both E-cadherin and vimentin positive, although the number of vimentin-positive tumour cells was much lower than in the vehicle-treated tumours (Fig. 6B). As with the primary resistant tumours, the two spindle cell AZD8931-resistant tumours had lost E-cadherin and were strongly vimentin positive. Strikingly, this was associated with loss of membranous HER2 expression (Fig. 6D,E). The remaining AZD8931-resistant tumour resembled the vehicle-treated tumours, being both E-cadherin and vimentin positive (Fig. 6C). Thus, the development of resistance to AZD8931 in a subpopulation of tumours is linked to increased expression of markers associated with the induction of EMT and loss of membranous HER2.

**Fig. 7.**
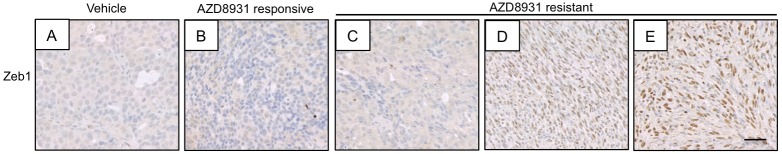
**Nuclear Zeb1 expression in spindle cell AZD8931-resistant tumours.** Immunohistochemical analysis of Zeb1 in (A) vehicle- and (B-E) AZD8931-treated tumours. (B) AZD8931-responsive tumour with growth inhibited by AZD8931. (C) AZD8931-resistant tumour that has retained the morphology of the AZD8931-naïve (vehicle) tumours. (D,E) AZD8931-resistant tumours with a spindle cell morphology. Scale bar: 50 µm.

In order to explore further the mechanisms of EMT induction in the resistant tumours, we looked at expression of Zeb1, which is a known transcriptional regulator of EMT. Upregulation of nuclear Zeb1 was seen only in the resistant spindle cell tumours, consistent with a Zeb1-regulated induction of EMT (Fig. 7).

## DISCUSSION

It is widely acknowledged that the use of conventional xenograft models for preclinical drug testing has limited predictive clinical value. Use of genetically engineered mouse models provides a useful alternative, in which drug response and resistance can be evaluated in situations that more faithfully recapitulate the human disease (Olive et al., 2009; Singh et al., 2010; Wang et al., 2012). Here, we show that the MMTV-NIC model has utility in assessing efficacy and resistance mechanisms of HER2-targeted therapies. Comparison of tumours with the same genotype demonstrated heterogeneity in the rate and extent of response to AZD8931, despite the use of predefined genetic mutations to drive tumour development. This variation in therapeutic response has been described previously in other genetically engineered models (Rottenberg et al., 2007) and contrasts with the more uniform response seen in xenograft studies (Becher and Holland, 2006). This is an important advance because it enables us more accurately to recapitulate the behaviour of human tumours and is most probably a result of the random acquisition of secondary mutations during tumour development and progression in these models. In addition, we show, for the first time, that acquired resistance to a HER2-targeted therapy can be modelled in MMTV-NIC tumours. The majority of studies exploring resistance to HER2-directed therapies have used cell-line-based approaches and, although numerous resistance mechanisms have been identified *in vitro*, clinical validation has proved challenging. The use of genetically engineered models to explore resistance mechanisms offers a more physiologically relevant system, with tumours developing resistance whilst exposed to ongoing *in vivo* selection pressures. Therefore, any resistance strategies identified might be more predictive of clinically relevant resistance mechanisms.

We identified PTEN loss as an important determinant of *de novo* AZD8931 resistance. Loss of PTEN and subsequent activation of the PI3K pathway has been identified as a key determinant of trastuzumab sensitivity and has been associated with poorer overall survival in trastuzumab-treated patients (Berns et al., 2007; Esteva et al., 2010; Nagata et al., 2004), although the impact on lapatinib resistance remains unclear (Xia et al., 2007). Like lapatinib, AZD8931 is a dual inhibitor of both HER2 and HER1; however, it has a unique profile of activity, being a more effective inhibitor of HER family signalling than lapatinib, resulting in a distinct profile of anti-tumour activity (Hickinson et al., 2010). Current trials are underway to determine whether the more effective simultaneous inhibition of HER family signalling provided by AZD8931 could have clinical benefit (Tjulandin et al., 2014); therefore, understanding potential mechanisms of resistance to AZD8931 is required. Both MMTV-NIC PTEN^+/+^ and MMTV-NIC PTEN^+/−^ tumours displayed reduced Akt activity after treatment with AZD8931, although in both models there was still evidence of residual activity following AZD8931 treatment. Incomplete inhibition of Akt signalling is a well-established mechanism of resistance to HER2-targeted therapies (Rexer and Arteaga, 2012) and is likely to contribute to the continued tumour progression in the MMTV-NIC PTEN^+/−^ model. In support of this, we saw no reduction in pAkt in AZD8931-resistant MMTV-NIC PTEN^+/−^ tumours following chronic drug treatment. Other studies have shown that combination therapy using a HER2 monoclonal antibody and an Akt inhibitor inhibited growth of MMTV-NIC PTEN^−/−^ tumours that were resistant to treatment with either drug alone (Wang et al., 2012). Taken together, this supports the use of Akt inhibitors in combination with HER2-targeted therapies, and a number of clinical trials are currently underway to evaluate the use of trastuzumab and/or lapatinib with Akt inhibitors.

One of the major advantages of using the MMTV-NIC tumours for modelling drug resistance was the generation of resistant tumours with distinct molecular phenotypes, which recapitulates in part the heterogeneity seen in the clinic. This provides a strong rationale for basing therapeutic decisions on the biology of the individual resistant tumour, which might be very different from that of the primary tumour. For example, the observation that a subset of our resistant tumours no longer expressed high levels of membranous HER2 could have a significant impact on future treatments. To date, major advances in overcoming clinical resistance to trastuzumab have focused on alternative strategies for targeting HER2 signalling, either by combining drugs that target different HER family receptors or through use of drug-antibody conjugates, such as trastuzumab-emtansine. As it is rarely mandatory to re-biopsy tumours at the time of entry into clinical trials, patients whose tumours no longer express HER2 risk being exposed to the toxicity of treatments that might not be anticipated to be effective.

Several *in vitro* studies have shown that resistance to lapatinib and trastuzumab is associated with induction of EMT (Creedon et al., 2014; Kim et al., 2013; Korkaya et al., 2012; Oliveras-Ferraros et al., 2012), and our finding that a subset of the resistant tumours have undergone EMT indicates that targeting pathways that regulate EMT might be effective in a subpopulation of resistant tumours (Singh and Settleman, 2010). Interestingly, the generation of mixed vimentin- and E-cadherin-positive tumours following re-implantation of an AZD8931-resistant tumour shows that the induction of EMT is not binary and that the tumours are highly plastic and can respond to microenvironmental factors that can affect their EMT status. The initial response of the re-implanted resistant tumour fragments to AZD8931 most probably reflects this plasticity, with the reversion to a more epithelial phenotype and the concomitant re-expression of HER2 conferring initial drug sensitivity. Although we have shown that the induction of EMT is associated with acquired resistance to AZD8931, induction of EMT in HER2-driven mouse mammary tumours via expression of an activating PI3K mutation was associated with *de novo* resistance to HER2-targeted therapies (Hanker et al., 2013), whereas *in vitro* studies showed that expression of transcription factors that drive EMT was causally related to *de novo* trastuzumab resistance (Oliveras-Ferraros et al., 2012).

One of the main challenges for the future is the identification of effective drug combinations to combat resistance to HER2-targeted therapies. Use of the transplantable tumour model described here provides a powerful preclinical tool with which to test potential novel drug combinations in resistant tumours, studies which to date rely on use of resistant cell lines established *in vitro*. For example, exploitation of the molecular differences in resistant tumours that have undergone EMT might provide alternative combination strategies for overcoming resistance in these tumours.

## MATERIALS AND METHODS

### Animals

MMTV-NIC mice expressing an oncogenic ErbB2/Neu allele and Cre recombinase under the control of the MMTV promoter were generated as previously described (Ursini-Siegel et al., 2008) and inter-crossed with floxed *Pten* (strain C;129S4-*Pten^tm1Hwu^*/J; Jackson Laboratory) mice to generate either MMTV-NIC PTEN^+/+^ or MMTV-NIC PTEN^+/−^ progeny on a FVB/N background. Genotyping was carried out by Transnetyx (Cordova, TN, USA). All experiments were conducted in compliance with UK Home Office guidelines. Nulliparous females were monitored twice weekly, using manual palpation, for tumour formation. The greatest tumour dimension and its perpendicular measurement were recorded, and when tumours had reached their maximal size (1.5 cm in one direction) as determined by Home Office regulations, mice were sacrificed. Tumours were then collected and fixed in 10% neutral buffered formalin. Tumours used for the generation of fragments were washed in ice-cold PBS and cut into 1 mm^3^ fragments and any macroscopic necrotic areas removed and then centrifuged at 450 ***g*** for 1 min. The supernatant, containing fibrous and necrotic material, was removed and the remaining fragments were suspended in cryopreservation buffer (50% Dulbecco's modified Eagle's medium, 45% fetal bovine serum and 5% dimethyl sulfoxide) and stored at −80°C. At the time of transplantation, fragments were defrosted at room temperature, washed in PBS and inserted into the fourth mammary fat pad.

For drug studies using AZD8931 (AstraZeneca Oncology iMed, Alderley Park, Macclesfield, UK), treatment was commenced when mice had at least one tumour ≥0.1 cm^3^ (index tumour) and continued until complete resolution of the index tumour or until the animal was sacrificed because of tumour size ≥1.5 cm (in any direction). Mice were dosed daily with vehicle (1% Tween 80 in PBS) or AZD8931 (100 mg/kg) suspended in 1% Tween 80 (in PBS) by oral gavage. To generate tumours that were resistant to AZD8931, an intermittent drug treatment schedule was performed. After transplantation of tumour fragments, tumours were allowed to grow to ≥0.1 cm^3^ before treatment with AZD8931 as above was started. When tumours regressed to <0.1 cm^3^, AZD8931 treatment was stopped and tumours were monitored twice weekly. If the tumour regrew, treatment was then restarted when tumours reached ≥0.3 cm^3^. This cycle was repeated until tumours developed resistance, and mice were sacrificed when the maximal tumour size (1.5 cm in any direction) was reached as permitted under UK Home Office regulations. For drug studies using paclitaxel, treatment was commenced when mice had at least one tumour ≥0.15 cm^3^ (index tumour) and continued until the animal was sacrificed because of tumour size or when the experiment was terminated 72 h after the fourth dose of paclitaxel. Mice were dosed weekly with vehicle (cremaphor EL:ethanol, 1:1, v:v) or paclitaxel (10 mg/kg) suspended in cremaphor EL:ethanol by intraperitoneal injection.

### Immunohistochemistry

Immunohistochemistry of formalin-fixed, paraffin-embedded tissues was performed as described previously (Karim et al., 2013). Primary antibodies used were as follows: HER2 (Invitrogen; catalogue no. AHO1011; 1:1000), pY1221/1222 HER2 (catalogue no. 2243; 1:400; Cell Signaling, UK), pY1289 HER3 (catalogue no. 4791; 1:100; Cell Signaling, UK), Ki67 (Vector; catalogue no. VP-RM04; 1:500), E-cadherin (catalogue no. 3195, 1:5000; Cell Signaling, UK), vimentin (catalogue no. 5741; 1:100; Cell Signaling, UK), pS473 Akt (catalogue no. 4060; 1:50; Cell Signaling, UK) and pT202/Y204 MAPK (catalogue no. 4370; 1:400; Cell Signaling, UK).

### Western blotting

Western blot analysis was performed as described previously (Karim et al., 2013). Primary antibodies used were HER2 (catalogue no. 2248; 1:1000; Cell Signaling, UK), pS473 Akt (catalogue no. 4060; 1:1000; Cell Signaling, UK), Akt (catalogue no. 9272; 1:1000; Cell Signaling, UK), PTEN (catalogue no. 9552; 1:1000; Cell Signaling, UK) and β-actin (catalogue no. A4700; Sigma, UK; 1:5000).

### Reverse-phase protein array analysis

Tumours were washed with PBS and lysed in 1% Triton X-100, 50 mM HEPES (pH 7.4), 150 mM sodium chloride, 1.5 mM magnesium chloride, 1 mM EGTA, 100 mM sodium fluoride, 10 mM sodium pyrophosphate, 1 mM sodium vanadate and 10% glycerol, supplemented with cOmplete ULTRA protease inhibitor and PhosSTOP phosphatase inhibitor cocktails (Sigma, UK). Cleared lysates were serially diluted to produce a dilution series comprising four serial twofold dilutions of each sample, which were spotted onto nitrocellulose-coated slides (Grace Bio-Labs, supplied by Sigma, UK) in triplicate in conditions of constant 70% humidity using the Aushon 2470 array platform (Aushon Biosystems, Billerica, MA, USA). Slides were hydrated in blocking buffer (Thermo Fisher Scientific, UK) and then incubated with primary antibodies (all 1:250; all from Cell Signaling, UK). Bound antibodies were detected by incubation with anti-rabbit DyLight 800-conjugated secondary antibody (New England BioLabs, UK). An InnoScan 710-IR scanner (Innopsys, Carbonne, France) was used to read the slides, and images were acquired at the highest gain without saturation of the fluorescence signal. The relative fluorescence intensity of each sample replicate was quantified using Mapix software (Innopsys).

The linear fit of the dilution series of each sample was determined for each primary antibody, from which median relative fluorescence intensities were calculated, and samples with *R*^2^<0.8 in all three replicates were excluded. Signal intensities were normalized by global sample median normalization (Guo et al., 2012).

## References

[DMM023143C1] Arteaga C. L., Sliwkowski M. X., Osborne C. K., Perez E. A., Puglisi F. and Gianni L. (2012). Treatment of HER2-positive breast cancer: current status and future perspectives. *Nat. Rev. Clin. Oncol.* 9, 16-32. 10.1038/nrclinonc.2011.177 22124364

[DMM023143C2] Becher O. J. and Holland E. C. (2006). Genetically engineered models have advantages over xenografts for preclinical studies. *Cancer Res.* 66, 3355-3359. 10.1158/0008-5472.CAN-05-3827 16585152

[DMM023143C3] Berns K., Horlings H. M., Hennessy B. T., Madiredjo M., Hijmans E. M., Beelen K., Linn S. C., Gonzalez-Angulo A. M., Stemke-Hale K., Hauptmann M. et al. (2007). A functional genetic approach identifies the PI3K pathway as a major determinant of trastuzumab resistance in breast cancer. *Cancer Cell* 12, 395-402. 10.1016/j.ccr.2007.08.030 17936563

[DMM023143C4] Creedon H., Byron A., Main J., Hayward L., Klinowska T. and Brunton V. G. (2014). Exploring mechanisms of acquired resistance to HER2 (human epidermal growth factor receptor 2)-targeted therapies in breast cancer. *Biochem. Soc. Trans.* 42, 822-830. 10.1042/BST20140109 25109964

[DMM023143C5] Esteva F. J., Guo H., Zhang S., Santa-Maria C., Stone S., Lanchbury J. S., Sahin A. A., Hortobagyi G. N. and Yu D. (2010). PTEN, PIK3CA, p-AKT, and p-p70S6K status: association with trastuzumab response and survival in patients with HER2-positive metastatic breast cancer. *Am. J. Pathol.* 177, 1647-1656. 10.2353/ajpath.2010.090885 20813970 PMC2947262

[DMM023143C6] Gajria D., Seidman A. and Dang C. (2010). Adjuvant taxanes: more to the story. *Clin. Breast Cancer* 10 Suppl. 2, S41-S49. 10.3816/CBC.2010.s.011 20805064

[DMM023143C7] Ghersi D., Wilcken N. and Simes R. J. (2005). A systematic review of taxane-containing regimens for metastatic breast cancer. *Br. J. Cancer* 93, 293-301. 10.1038/sj.bjc.6602680 16052223 PMC2361568

[DMM023143C8] Guo H., Liu W., Ju Z., Tamboli P., Jonasch E., Mills G. B., Lu Y., Hennessy B. T. and Tsavachidou D. (2012). An efficient procedure for protein extraction from formalin-fixed, paraffin-embedded tissues for reverse phase protein arrays. *Proteome Sci.* 10, 56 10.1186/1477-5956-10-56 23006314 PMC3561137

[DMM023143C9] Hanker A. B., Pfefferle A. D., Balko J. M., Kuba M. G., Young C. D., Sanchez V., Sutton C. R., Cheng H., Perou C. M., Zhao J. J. et al. (2013). Mutant PIK3CA accelerates HER2-driven transgenic mammary tumors and induces resistance to combinations of anti-HER2 therapies. *Proc. Natl. Acad. Sci. USA* 110, 14372-14377. 10.1073/pnas.1303204110 23940356 PMC3761610

[DMM023143C10] Hickinson D. M., Klinowska T., Speake G., Vincent J., Trigwell C., Anderton J., Beck S., Marshall G., Davenport S., Callis R. et al. (2010). AZD8931, an equipotent, reversible inhibitor of signaling by epidermal growth factor receptor, ERBB2 (HER2), and ERBB3: a unique agent for simultaneous ERBB receptor blockade in cancer. *Clin. Cancer Res.* 16, 1159-1169. 10.1158/1078-0432.CCR-09-2353 20145185

[DMM023143C11] Karim S. A., Creedon H., Patel H., Carragher N. O., Morton J. P., Muller W. J., Evans T. R. J., Gusterson B., Sansom O. J. and Brunton V. G. (2013). Dasatinib inhibits mammary tumour development in a genetically engineered mouse model. *J. Pathol.* 230, 430-440. 10.1002/path.4202 23616343

[DMM023143C12] Kim H.-P., Han S.-W., Song S.-H., Jeong E.-G., Lee M.-Y., Hwang D., Im S.-A., Bang Y.-J. and Kim T.-Y. (2013). Testican-1-mediated epithelial-mesenchymal transition signaling confers acquired resistance to lapatinib in HER2-positive gastric cancer. *Oncogene* 33, 3334-3341. 10.1038/onc.2013.285 23873022

[DMM023143C13] Korkaya H., Kim G.-I., Davis A., Malik F., Henry N. L., Ithimakin S., Quraishi A. A., Tawakkol N., D'Angelo R., Paulson A. K. et al. (2012). Activation of an IL6 inflammatory loop mediates trastuzumab resistance in HER2+ breast cancer by expanding the cancer stem cell population. *Mol. Cell* 47, 570-584. 10.1016/j.molcel.2012.06.014 22819326 PMC3432419

[DMM023143C14] Nagata Y., Lan K.-H., Zhou X., Tan M., Esteva F. J., Sahin A. A., Klos K. S., Li P., Monia B. P., Nguyen N. T. et al. (2004). PTEN activation contributes to tumor inhibition by trastuzumab, and loss of PTEN predicts trastuzumab resistance in patients. *Cancer Cell* 6, 117-127. 10.1016/j.ccr.2004.06.022 15324695

[DMM023143C15] Olive K. P., Jacobetz M. A., Davidson C. J., Gopinathan A., McIntyre D., Honess D., Madhu B., Goldgraben M. A., Caldwell M. E., Allard D. et al. (2009). Inhibition of Hedgehog signaling enhances delivery of chemotherapy in a mouse model of pancreatic cancer. *Science* 324, 1457-1461. 10.1126/science.1171362 19460966 PMC2998180

[DMM023143C16] Oliveras-Ferraros C., Corominas-Faja B., Cufi S., Vazquez-Martin A., Martin-Castillo B., Iglesias J. M., Lopez-Bonet E., Martin A. G. and Menendez J. A. (2012). Epithelial-to-mesenchymal transition (EMT) confers primary resistance to trastuzumab (Herceptin). *Cell Cycle* 11, 4020-4032. 10.4161/cc.22225 22992620 PMC3507497

[DMM023143C17] Rexer B. N. and Arteaga C. L. (2012). Intrinsic and acquired resistance to HER2-targeted therapies in HER2 gene-amplified breast cancer: mechanisms and clinical implications. *Crit. Rev. Oncog.* 17, 1-16. 10.1615/CritRevOncog.v17.i1.20 22471661 PMC3394454

[DMM023143C18] Rottenberg S., Nygren A. O. H., Pajic M., van Leeuwen F. W. B., van der Heijden I., van de Wetering K., Liu X., de Visser K. E., Gilhuijs K. G., van Tellingen O. et al. (2007). Selective induction of chemotherapy resistance of mammary tumors in a conditional mouse model for hereditary breast cancer. *Proc. Natl. Acad. Sci. USA* 104, 12117-12122. 10.1073/pnas.0702955104 17626183 PMC1914039

[DMM023143C19] Schade B., Rao T., Dourdin N., Lesurf R., Hallett M., Cardiff R. D. and Muller W. J. (2009). PTEN deficiency in a luminal ErbB-2 mouse model results in dramatic acceleration of mammary tumorigenesis and metastasis. *J. Biol. Chem.* 284, 19018-19026. 10.1074/jbc.M109.018937 19435886 PMC2707232

[DMM023143C20] Sharpless N. E. and DePinho R. A. (2006). The mighty mouse: genetically engineered mouse models in cancer drug development. *Nat. Rev. Drug Discov.* 5, 741-754. 10.1038/nrd2110 16915232

[DMM023143C21] Singh A. and Settleman J. (2010). EMT, cancer stem cells and drug resistance: an emerging axis of evil in the war on cancer. *Oncogene* 29, 4741-4751. 10.1038/onc.2010.215 20531305 PMC3176718

[DMM023143C22] Singh M., Lima A., Molina R., Hamilton P., Clermont A. C., Devasthali V., Thompson J. D., Cheng J. H., Bou Reslan H., Ho C. C. K. et al. (2010). Assessing therapeutic responses in Kras mutant cancers using genetically engineered mouse models. *Nat. Biotech.* 28, 585-593. 10.1038/nbt.1640 20495549

[DMM023143C23] Tjulandin S., Moiseyenko V., Semiglazov V., Manikhas G., Learoyd M., Saunders A., Stuart M. and Keilholz U. (2014). Phase I, dose-finding study of AZD8931, an inhibitor of EGFR (erbB1), HER2 (erbB2) and HER3 (erbB3) signaling, in patients with advanced solid tumors. *Invest. New Drugs* 32, 145-153. 10.1007/s10637-013-9963-6 23589215

[DMM023143C24] Ursini-Siegel J., Hardy W. R., Zuo D., Lam S. H. L., Sanguin-Gendreau V., Cardiff R. D., Pawson T. and Muller W. J. (2008). ShcA signalling is essential for tumour progression in mouse models of human breast cancer. *EMBO J.* 27, 910-920. 10.1038/emboj.2008.22 18273058 PMC2274931

[DMM023143C25] van Miltenburg M. H. and Jonkers J. (2012). Using genetically engineered mouse models to validate candidate cancer genes and test new therapeutic approaches. *Curr. Opin. Genet. Dev.* 22, 21-27. 10.1016/j.gde.2012.01.004 22321988

[DMM023143C26] Wang Q., Li S.-H., Wang H., Xiao Y., Sahin O., Brady S. W., Li P., Ge H., Jaffee E. M., Muller W. J. et al. (2012). Concomitant targeting of tumor cells and induction of T-cell response synergizes to effectively inhibit trastuzumab-resistant breast cancer. *Cancer Res.* 72, 4417-4428. 10.1158/0008-5472.CAN-12-1339-T 22773664 PMC3556997

[DMM023143C27] Xia W., Husain I., Liu L., Bacus S., Saini S., Spohn J., Pry K., Westlund R., Stein S. H. and Spector N. L. (2007). Lapatinib antitumor activity is not dependent upon phosphatase and tensin homologue deleted on chromosome 10 in ErbB2-overexpressing breast cancers. *Cancer Res.* 67, 1170-1175. 10.1158/0008-5472.CAN-06-2101 17283152

